# Author Correction: In-situ formation of one-dimensional coordination polymers in molecular junctions

**DOI:** 10.1038/s41467-021-24204-7

**Published:** 2021-06-28

**Authors:** Anton Vladyka, Mickael L. Perrin, Jan Overbeck, Rubén R. Ferradás, Víctor García-Suárez, Markus Gantenbein, Jan Brunner, Marcel Mayor, Jaime Ferrer, Michel Calame

**Affiliations:** 1 Department of Physics, University of Basel, Klingelbergstrasse 82, CH-4056 Basel, Switzerland; 2 Empa, Swiss Federal Laboratories for Materials Science and Technology, Überlandstrasse 129, CH-8600 Dübendorf, Switzerland; 3 Swiss Nanoscience Institute, Klingelbergstrasse 82, CH-4056 Basel, Switzerland; 4 Department of Physics, University of Oviedo, 33007 Oviedo, Spain; 5 Laboratoire de Chimie et Physique Quantiques, IRSAMC, Université Toulouse III - Paul Sabatier, CNRS, 118 Route de la Narbone, 31062 Toulouse Cedex, France; 6 Nanomaterials and Nanotechnology Research Center, CSIC - Universidad de Oviedo, 33007 Oviedo, Spain; 7 Department of Chemistry, University of Basel, Klingelbergstrasse 82, CH-4056 Basel, Switzerland; 8 Institute for Nanotechnology (INT), Karlsruhe Institute of Technology (KIT), P. O. Box 3640, 76021 Karlsruhe, Germany; 9 Lehn Institute of Functional Materials (LFM), Sun Yat Sen University (SYSU), XinGangXi Rd. 135, 510275 Guangzhou, P. R. China

**Keywords:** Electronic devices, Molecular electronics

Correction to: *Nature Communications* 10.1038/s41467-018-08025-9, published online 16 January 2019.

The original version of this Article omitted the following from the Acknowledgements:

R. R. F., V. G. S. and J. F. have also been funded by the Spanish Ministerio de Economía y Competitividad and Ministerio de Ciencia e Innovación (grants no. FIS2012-34858 and FIS2015-63918-R, respectively).

This has now been corrected in both the PDF and HTML versions of the Article.

