# Supplementary material for: In-situ formation of one-dimensional coordination polymers in molecular junctions
Source: Nat Commun. 2019 Jan 16;10:262. doi: 10.1038/s41467-018-08025-9 (PMC6335403; doi:10.1038/s41467-018-08025-9)
Supplement: Supplementary file 1 — Supplementary Information [file 41467_2018_8025_MOESM1_ESM.pdf]

Supporting Information
for
In-situ formation of one-dimensional coordination polymers in molecular junctions

Anton Vladyka,[1,2] Mickael Perrin,[2] Jan Overbeck,[1,2,3] Rubén Ferradás,[4,5] Víctor García-Suárez,[4,6] Markus Gantenbein,[7] Jan Brunner,[1] Marcel Mayor,[7,8,9] Jaime Ferrer,[4,6] and Michel Calame[1,2,3]

[1]*Department of Physics, University of Basel, Klingelbergstrasse 82, CH-4056, Basel, Switzerland*

[2]*Empa, Swiss Federal Laboratories for Materials Science and Technology, Überlandstrasse 129, CH-8600 Dübendorf, Switzerland*

[3]*Swiss Nanoscience Institute, Klingelbergstrasse 82, CH-4056, Basel, Switzerland*

[4]*Department of Physics, University of Oviedo, Spain*

[5]*Laboratoire de Chimie et Physique Quantiques, IRSAMC, Université Toulouse III - Paul Sabatier, CNRS, 118 Route de la Narbone, 31062 Toulouse Cedex, France*

[6]*Nanomaterials and Nanotechnology Research Center, CSIC & Universidad de Oviedo, Spain*

[7]*Department of Chemistry, University of Basel, Klingelbergstrasse 82, CH-4056, Basel, Switzerland*

[8]*Institute for Nanotechnology (INT), Karlsruhe Institute of Technology (KIT), P. O. Box 3640, 76021 Karlsruhe, Germany*

[9]*Lehn Institute of Functional Materials (LFM), Sun Yat Sen University (SYSU), XinGangXi Rd. 135, 510275 Guangzhou (P. R. China)*

Contents

Supplementary Note 1: Yield estimation 3

Supplementary Note 2: Plateau length 5

Supplementary Note 3: Trimer detection scheme 6

Supplementary Note 4: Plateau shape analysis 7

Supplementary Note 5: Fluctuation analysis 9

Supplementary Note 6: BdNC junction formation in dry conditions 11

Supplementary Note 7: DFT calculations: Methodology 12

Supplementary Note 8: Estimates of gold-molecule binding affinities via total energy calculations 13

Supplementary Note 9: Molecular Dynamics simulations of opening, closing and closing-opening cycles 15

Supplementary Note 10: Chaining effects 17

Supplementary Note 11: Scissor corrections scheme 20

Supplementary Note 12: Statistics of the opening cycles 22

Supplementary Note 13: Decay constant for a chain with two interconnecting gold atoms 23

Supplementary References 24

Supplementary Note 1: Yield estimation

The yield of the molecular junction formation was obtained as follows: 1) First, for each conductance peak the peak range was determined based on the two local minima around the peak. 2) The length of each trace within the corresponding conductance range was extracted for the measurements with molecules, as well as for the empty tunneling junctions. 3) The trace length distribution was fitted to Gaussian distributions. 4) The yield of molecules is defined as the area below the molecular curve, minus it's intersect with the solvent curve (Supp. Fig. 1). Analysis of the yields for all plateaus is shown in Supplementary Figure 1b–g, with the obtained yields and fit parameters summarized in Supplementary Table I.

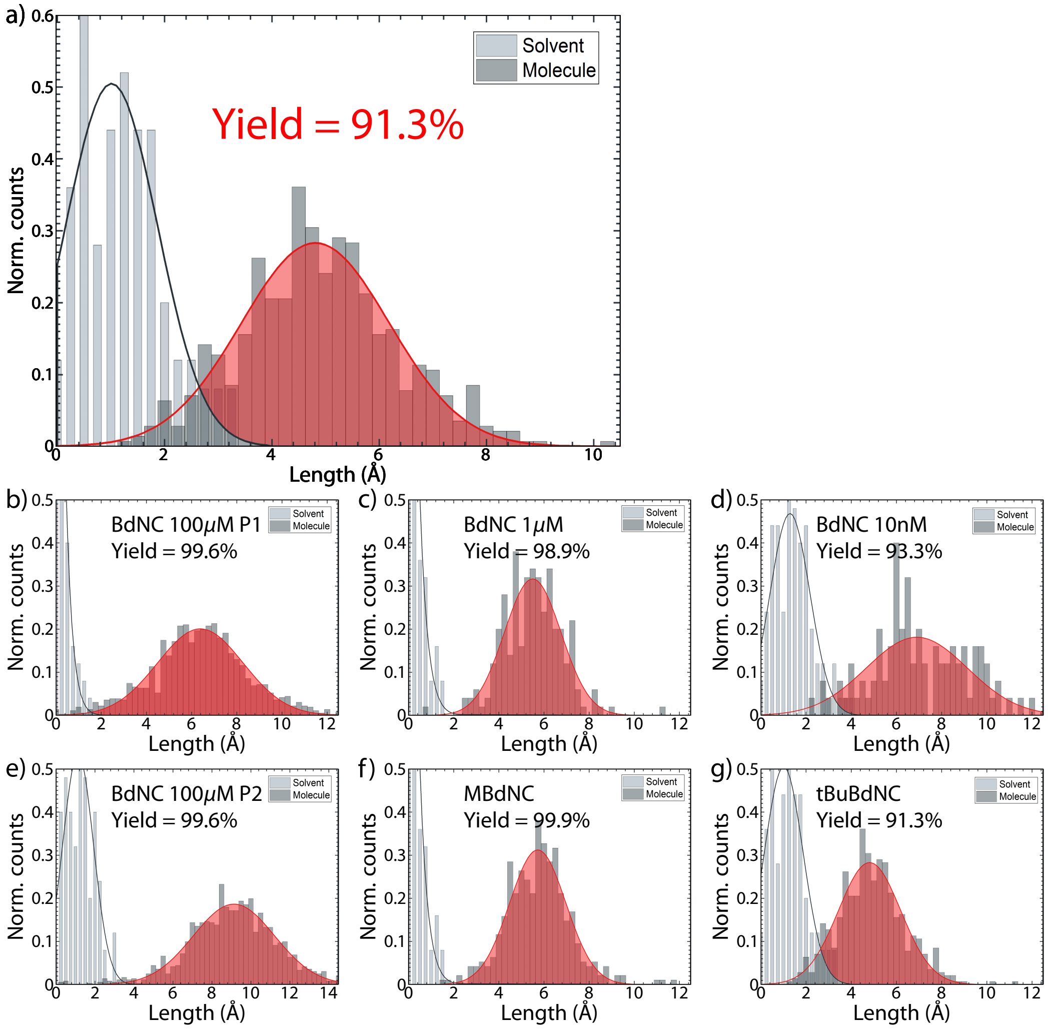

Supplementary Figure 1: Yield of junction formation estimation. (a) Scheme of the plateau formation yield estimation. The histograms represent the trace length distribution, colored light gray for solvent and dark gray for molecules. The black and red curves are Gaussian fits of the histograms. Counts are normalized by bin size and number of traces in the dataset, such that the area below the corresponding Gaussian curve is unity. We define the yield as the area below the molecular curve minus its intersection with the area below the solvent curve (filled area). (b-g) Yield of plateau formation for all measurements.

Supplementary Table I: Yield of junction formation parameters

Plateau	$\log G_1$	$\log G_2$	Yield (%)
BdNC 100 µM P1	−1.3	−3.0	99.6
BdNC 100 µM P2	−3.0	−4.7	99.6
BdNC 1 µM	−1.6	−3.3	98.9
BdNC 1 nM	−2.1	−4.9	93.3
MBdNC 100 µM	−1.6	−3.3	99.9
tBuBdNC 100 µM	−2.2	−4.7	91.3

Supplementary Note 2: Plateau length

For the 100 µM BdNC measurements, the plateau length was defined as the length of the conductance trace within a specified conductance window, see Supp. Fig. 2. The ranges were defined as follows: $(10^{-3.0}\,\mathrm{G_0}, 10^{-1.3}\,\mathrm{G_0})$ for P1 plateau and $(10^{-4.7}\,\mathrm{G_0}, 10^{-3.0}\,\mathrm{G_0})$ for P2 plateau. By fitting the plateau length distributions to Gaussian functions, we obtained the following average plateau lengths: $L_{\mathrm{P1}} = 6.4\,\text{Å}$ and $L_{\mathrm{P2}} = 9.0\,\text{Å}$ for P1 and P2 plateau, respectively.

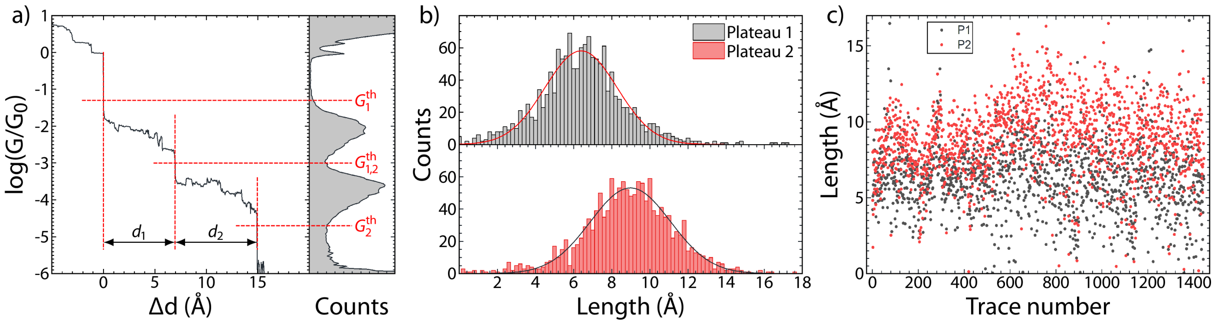

Supplementary Figure 2: (a) Plateau length analysis procedure. (b) Distribution of the obtained plateau lengths. Black and red curves depict a fit to a Gaussian distribution, yielding an average plateau length of 6.4 Å and 9.0 Å for P1 and P2 plateau, respectively. (c) Scatter plot of the plateau length.

Supplementary Note 3: Trimer detection scheme

To analyze the formation of the third plateau (P3), *i.e.*, the chain formed by three monomer BdNC units, we performed the same procedure as for plateau length analysis. Assuming exponential decay of the conductance with molecular length, the expected conductance value for P3 plateau is about $10^{-5.4}\,\mathrm{G_0} = 2.5 \cdot 10^{-6}\,\mathrm{G_0}$. We therefore performed the plateau length analysis for a conductance range of $(10^{-5.7}\,\mathrm{G_0}, 10^{-5.0}\,\mathrm{G_0})$. The distribution of trace length is this conductance range shown in Supp. Fig. 3a. Using $L_{\mathrm{th}} = 1.7\,\text{Å}$ as selection criteria, we extracted 420 traces (29%) with three conductance plateaus (see main text).

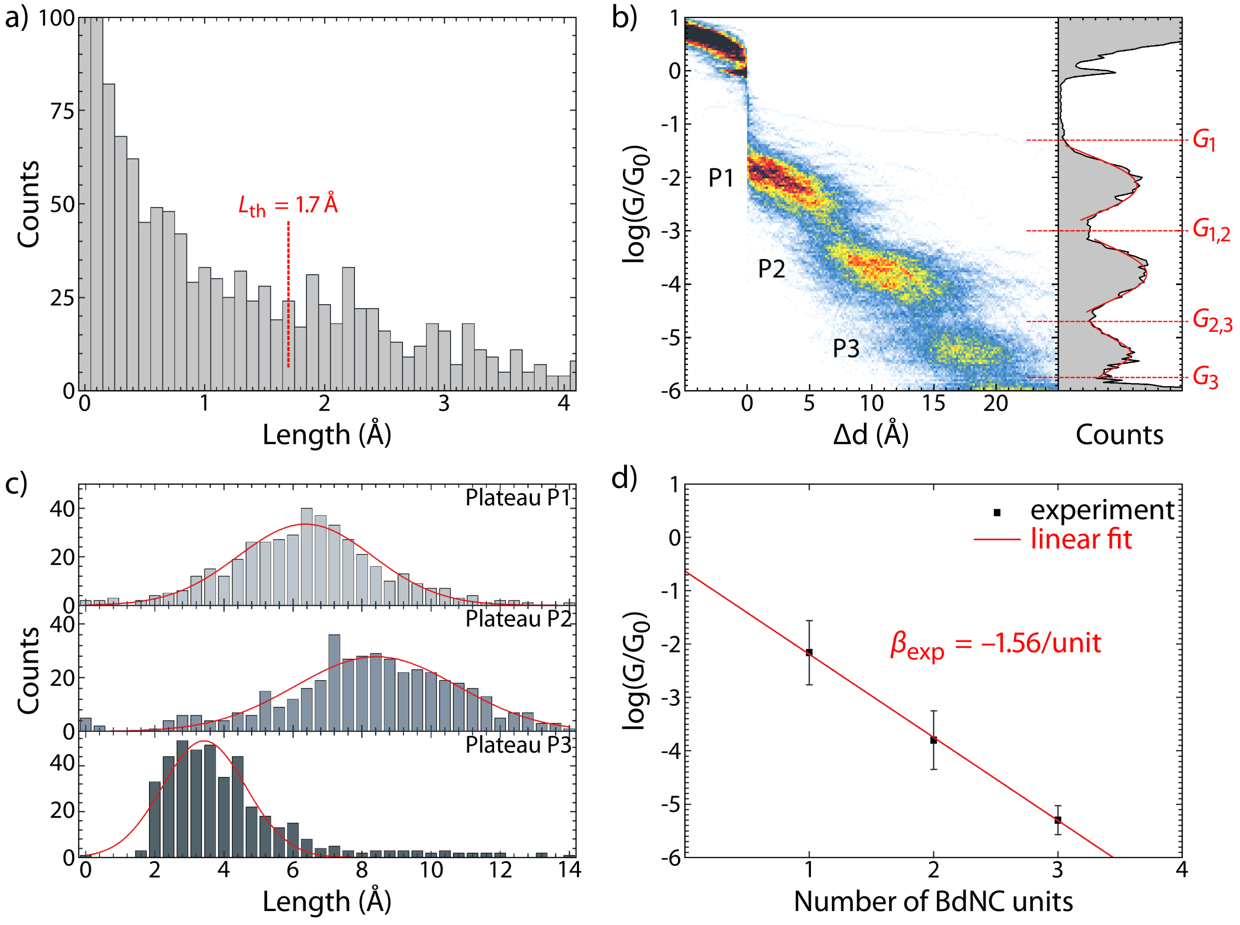

Supplementary Figure 3: (a) Distribution of the trace length in the conductance range of $(10^{-5.7}\,\mathrm{G_0}, 10^{-5.1}\,\mathrm{G_0})$. Red dashed line depicts the lengths threshold used as a selection criterium. (b) Combined 2D/1D histogram for selected 420 conductance traces which exhibit third plateau. Horizontal dashed lines represent the conductance thresholds for each plateau: $G_1 = 10^{-1.3}\,\mathrm{G_0}$, $G_{1,2} = 10^{-3.0}\,\mathrm{G_0}$, $G_{2,3} = 10^{-4.7}\,\mathrm{G_0}$, $G_3 = 10^{-5.75}\,\mathrm{G_0}$. (c) Plateau length analysis for each plateau. Red curves depict fitting the distributions to Gaussian, yielding plateau length of 6.4 Å, 8.4 Å and 3.4 Å for plateaus P1, P2 and P3, respectively. (d) Conductance decay as a function of the number of BdNC units in a chain.

Supplementary Note 4: Plateau shape analysis

For the plateau shape analysis, only the conductance-displacement histogram data in the conductance range around the plateau is considered. The conductance plateaus are fitted

to the bivariate Gaussian distribution:

$$N \sim \exp\left(-\frac{((x-x_0)\cos\phi-(y-y_0)\sin\phi)^2}{2\sigma_x^2} - \frac{((x-x_0)\sin\phi+(y-y_0)\cos\phi)^2}{2\sigma_y^2}\right) \quad (1)$$

Plateau shape analysis for all measurements is shown in Figure 4, and all deduced plateau shape parameters are summarized in Supplementary Table II.

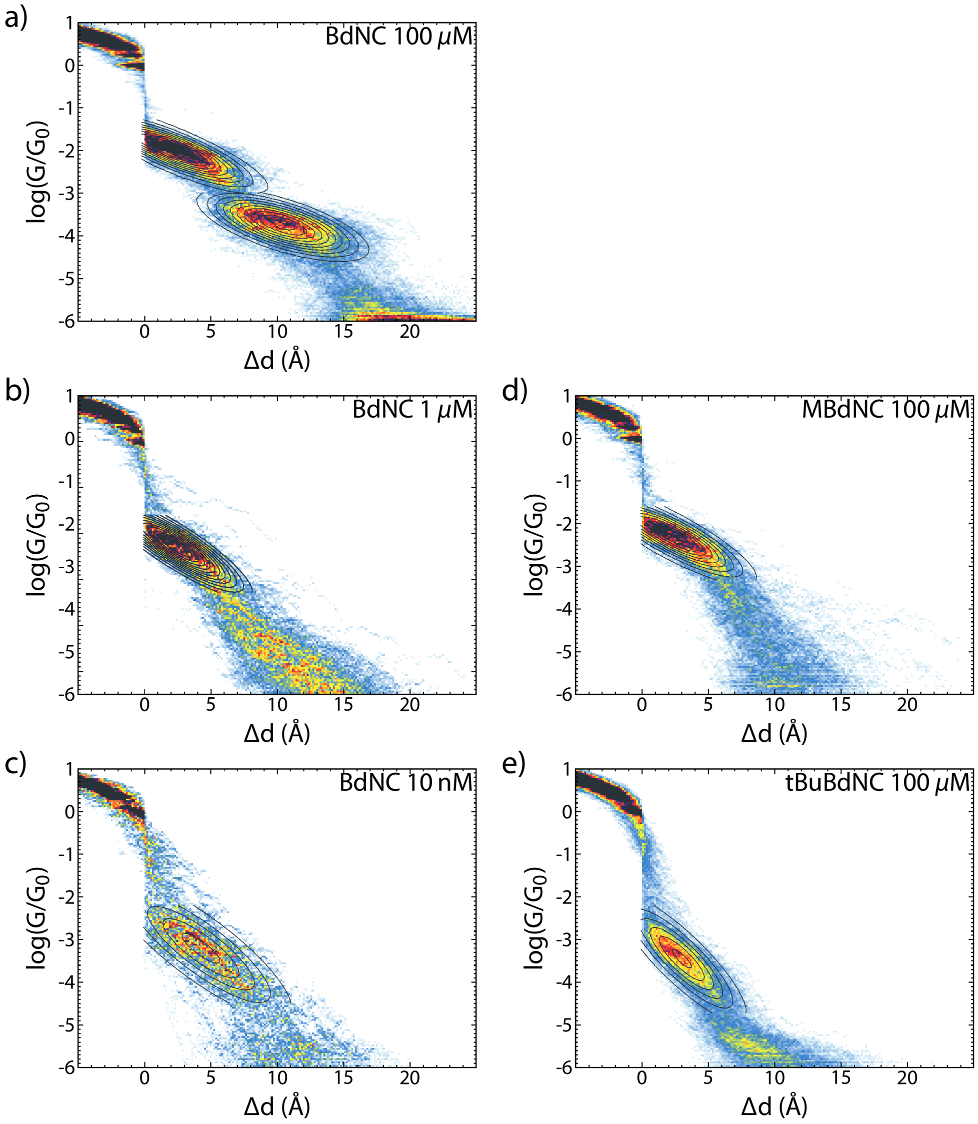

Supplementary Figure 4: Plateau shape analysis for all measurements.

Supplementary Table II: Shape parameters of the conductance plateaus

Measurement	x_0	σ_x	y_0	σ_y	ϕ
BdNC 10 nM	4.0	3.9	−3.2	0.43	0.21
BdNC 1 µM	2.5	3.0	−2.3	0.27	0.165
BdNC 100 µM P1	2.1	3.4	−2.0	0.24	0.115
MBdNC 100 µM	2.3	3.0	−2.3	0.27	0.154
tBuBdNC 100 µM	3.0	2.6	−3.4	0.37	0.26
BdNC 100 µM P2	10.6	3.5	−3.7	0.34	0.093

In the main text we refer to the parameter $y_0 \equiv \log(G_C/G_0)$ as the center of mass of the conductance plateau, σ_y as the plateau width, and $\tan\phi$ as the slope.

Supplementary Note 5: Fluctuation analysis

For quantitative characterization of the conductance fluctuations within the plateaus, we performed a linear fit of the conductance trace ($\bar{g}(\Delta d)$ within the conductance plateau range (Supp. Fig. 5a). This was done was each traces of all datasets (100 µM, 1 µM, 1 nM solutions of BdNC, 100 µM solutions of MBdNC and tBuBdNC). Then, within the conductance plateau range we calculated for every trace the fluctuations of the conductance around the linear fit of the trace, *i.e.*, if $g(\Delta d)$ is a conductance trace and $\hat{g}(\Delta d) = \hat{g}_1 - \hat{g}_2 \cdot \Delta d$ is a linear fit of $g(\Delta d)$ within the plateau, the fluctuations f are defined as $f \equiv \sigma\left[g(\Delta d) - \hat{g}(\Delta d)\right]$. The distributions of σ per dataset are then fitted to Gaussian distribution in order to obtained the 'average' of the fluctuations, which are presented in Table III, alongside their standard deviations.

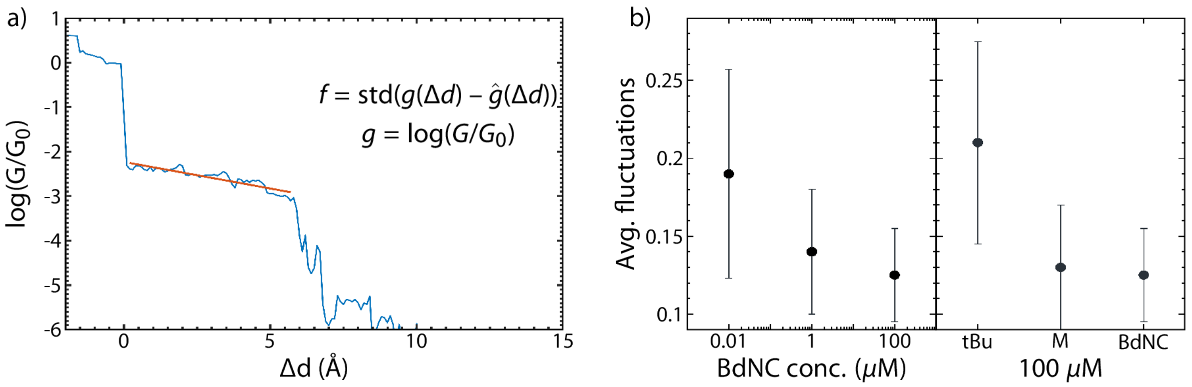

Supplementary Figure 5: (a) Example conductance trace ($g(\Delta d)$, blue) for MBdNC molecule and the corresponding linear fit ($\hat{g}(\Delta d)$, orange) of the trace within the conductance plateau. (b) Calculated average fluctuations for all measured compounds.

Supplementary Table III: Parameters of the distributions

Name of measurements	μ	σ
BdNC 100 µM	0.128	0.03
MBdNC 100 µM	0.133	0.04
tBuBdNC 100 µM	0.207	0.06
BdNC 1 µM	0.14	0.04
BdNC 10 nM	0.19	0.67

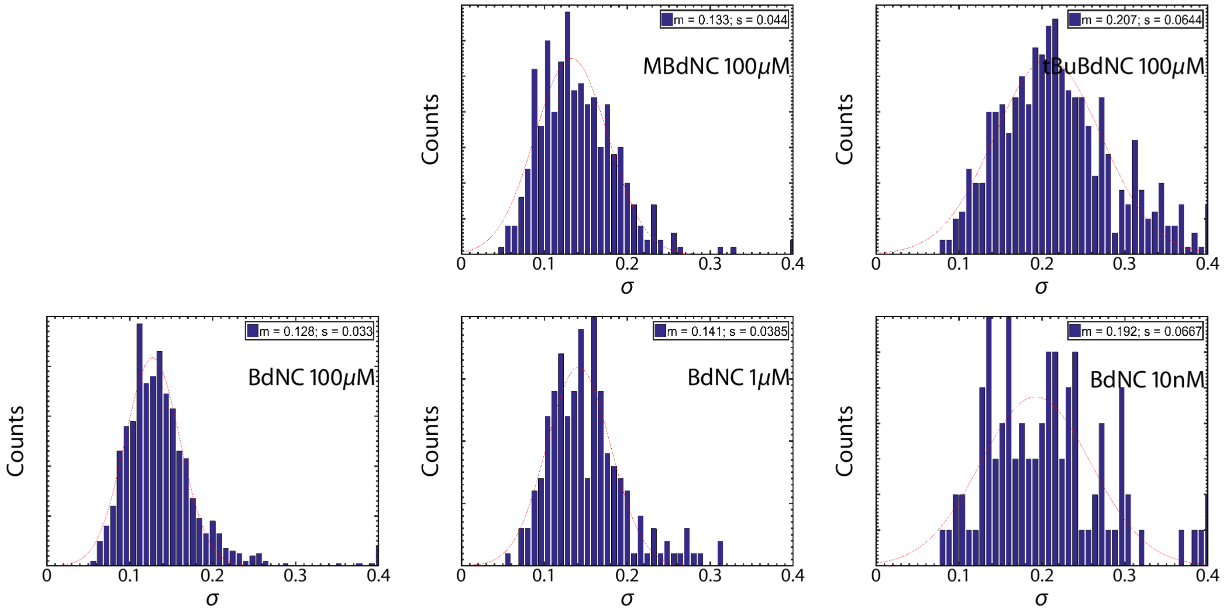

Supplementary Figure 6: Distributions of σ values for each dataset.

With the decrease of concentration and increase of the side group, the fluctuations of conductance within conductance plateau increase, following the same trend as the width of the plateau in 2D plateaus shape analysis.

Supplementary Note 6: BdNC junction formation in dry conditions

Supplementary Figure 7 presents a conductance-displacement histogram on BdNC performed in dry condition using the same settings as in the main text. As this sample belongs to a different batch and the attenuation factor has not been calibrated, the x-axis corresponds to the linear displacement of the stepper motor in microns instead of the actual electrode displacement in Ångstroms.

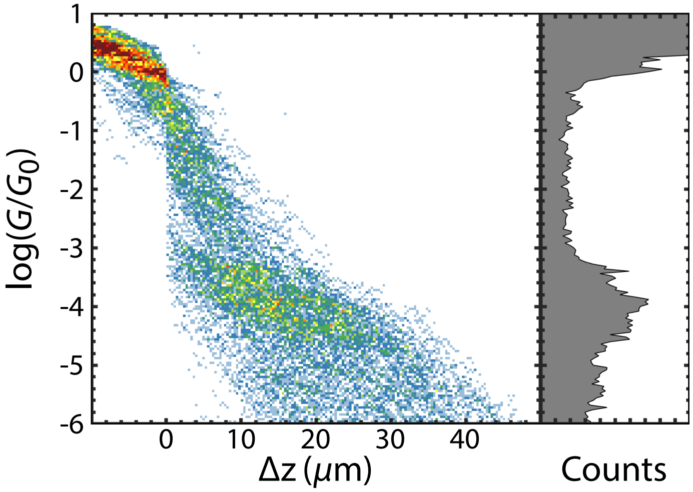

Supplementary Figure 7: Combined 2D-1D conductance histogram of a measurement performed in dry conditions using similar measurement settings as in the main text.

Supplementary Note 7: DFT calculations: Methodology

We have simulated a variety of gold-molecule-gold junctions using the *ab-initio* Molecular Dynamics simulation package SIESTA[1] to shed theoretical light upon our experiments. Here, a molecule means Benzene diiso-cyano (BdNC), Tertbutyl Benzene diiso-cyano (tBuBdNC), Benzene Di-cyano (BdCN) and Benzene dithiol (BDT), and gold means some sort of gold electrode as described below. We have used a double-ζ polarized basis set for each atom in the different molecules and a modified single-ζ polarized (s+p+d) for those gold atoms belonging in the bulk part of the electrodes. Gold atoms at the electrodes surfaces and at the pyramids have been supplemented with diffuse s-orbitals. We have used long radii for the different basis orbitals that provide accurate results for the binding energies of diverse molecules on gold (111) surfaces[2]. To check for exchange-correlation functionals, we have computed the total energies of simplified junctions where the electrodes consisted of 3-1 gold pyramids each followed by vacuum. We have computed the total energy of these junctions as a function of the molecule-tip distance for LDA[3], GGA[4] and the DRSLL van der Waals[5] functionals, and found that for each molecule, the curves for the three functionals roughly fall onto each other. We have then used GGA to carry out more detailed calculations of the binding energies of the different molecules to a single flat (111) gold surface. We have also simulated junctions where the electrodes were just gold chains to carry out complementary

checks of total energies.

We have carried out an extensive set of ab-initio room-temperature molecular dynamics simulations, using more realistic electrodes. They consisted of flat (111) surfaces, to which pyramids where attached. These pyramids had five gold atomic layers and were terminated by a single gold atom, as shown in Figure 2b in the main text. These molecular dynamics calculations were performed using an LDA exchange-correlation functional and a Nose Thermostat. We have estimated the differential conductance of the molecular junctions using the quantum transport code GOLLUM[6]. We have tested the validity of the phenomenological Scissors Correction scheme for our junctions, as discussed below.

Supplementary Note 8: Estimates of gold-molecule binding affinities via total energy calculations

We have performed several complementary calculations to identify the binding affinities between each of the above molecules and gold at low molecule concentrations. We have first attached a single BdNC, BdCN or BdT molecule to the tips of two 3-1 gold pyramids simulating a simplified junction. We have separated the two tips from the molecule symmetrically and computed the total energy per tip as a function of the distance between the tip gold atoms and the terminating atoms at the molecule. These curves are plotted in the top panel in Supp. Fig. 8. The energy curves over-estimate the binding energies of the molecule-gold bonds[2] because of the simplified junction geometry, but provide a first intuition of the affinity of those molecules for gold. The figure shows that the binding energy of BdNC is considerably larger than BDT, giving a first indication that BdNC molecules show the strongest affinity for gold among all considered molecules. To identify how each molecule arranges itself on top of gold at low molecule coverage, we have performed total energy calculations of a single gold (111) flat surface. We have oriented the molecules at a given angle with respect to the gold surface and then increased the surface-molecule distance.

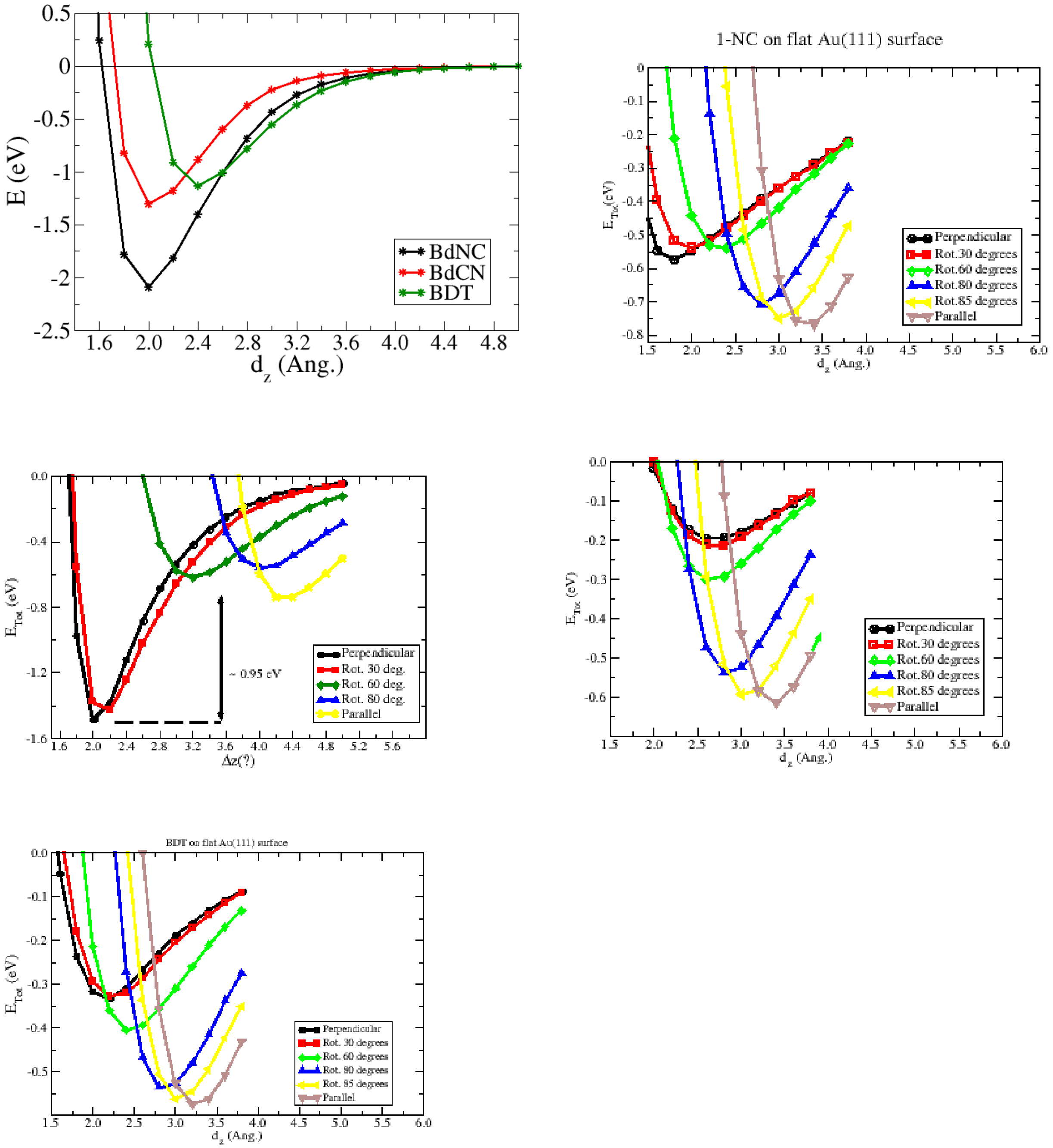

Supplementary Figure 8: (top) Energy of junctions consisting of a single BdNC (black), BdCN (red) and BDT (green) molecules bridging two 3-1 gold tips as a function of the tip-molecule distance. The total energy is divided by two to estimate the binding energy per tip. (Middle and bottom) Total energy of BdNC (middle-left), tBu-BdNC (middle-right), BdCN (bottom-left) and BDT (bottom-right) molecules on top of a flat (111) gold surface, as a function of the surface-molecule distance. Each curve represents a different orientation of the molecule axis relative to the flat surface, measured in terms of the relative angle.

We have collated the energy-distance curves for different angles in the same plot for each molecule. These are shown in the middle and bottom panels in Figure 8. The curves demonstrate that tBuBdNC molecules have a strong tendency to orient themselves perpendicular to the surface. The plot for BdNC molecules is similar in what it displays two minima corresponding to zero and ninety degrees relative orientations. Although the deepest minimum corresponds now to BdNC lying flat on the surface, the shallower minimum indicates that BdNC may also orient perpendicular to the surface. In contrast, we find that BdCN and BDT molecules show no traces of a minimum at a perpendicular orientation, so that these molecules will just tend to lie flat on the gold surface at low coverages. The envelope of each of those figures is plotted in Figure 2 in the main text.

Supplementary Note 9: Molecular Dynamics simulations of opening, closing and closing-opening cycles

We have performed a sizeable number of opening, closing and closing-opening cycles for BdNC molecules, and a few of them also for tBu-BDNC, BDT and BDCN, where each of the electrodes is pulled apart or pushed together in length-steps of 0.1 Å. We have performed ab-initio room-temperature molecular Dynamics (RTMD) simulations of different sorts in between every length-step until we have achieved simulations that look reasonable enough. We have followed an approach similar to reference[7], although we have had to increase the number of MD steps to reach the needed stability of the junctions. In our first attempt, we have taken 60 ab-initio RTMD time-step of 1 fs each every length-step, that corresponds to a speed of 300 m/s. We have found that the pyramids and the first (111) gold layer become destroyed after a few length steps, whereby several gold atoms are pulled away and one or two gold filaments are formed. In retrospective, we have understood that this behavior is an artefact caused by the speed of the theory cycles, that shakes atoms too strongly. As a comparison we note that the order of magnitude of the experimental speed of a cycle can easily be obtained by assuming that pulling a distance of, say, 20 Å takes about 20 s. This yields a speed of 10^{-10} m/s, that is many orders of magnitude more adiabatic than the above-described theory cycle. To achieve better adiabaticity, we have followed a second procedure for every length-step: we have relaxed forces taking 60 force-relaxation steps right after each length-step, followed by 60 ab-initio RTMD time-steps of 1 fs each. We

have found that the pyramids are now stable, and that filament formation is depleted. We have found that the quality of the ensuing MD simulations enables us to draw conclusions about the structural properties of the junctions. However, we have found that the quantum transport results are cleaner if we follow a third more careful procedure. Here, we start by placing the two electrodes far enough apart, and place a molecule or complex attached to one of the two pyramid tips. We then execute 1500 ab-initio RTMD time-steps of 1 fs each to relax the geometry. We then start a full closing - opening cycle, were we take 200 ab-initio RTMD time-steps of 1 fs in between every two length-steps. We start merging the electrodes together until the single-molecule junction is formed, keep pushing until a gold neck is formed and beyond. We then start to pull the electrodes apart until the neck is broken and keep pulling until the junction is broken. We have found that this procedure delivers simulations that look much more adiabatic, and that the quantum transport results look reasonable enough.

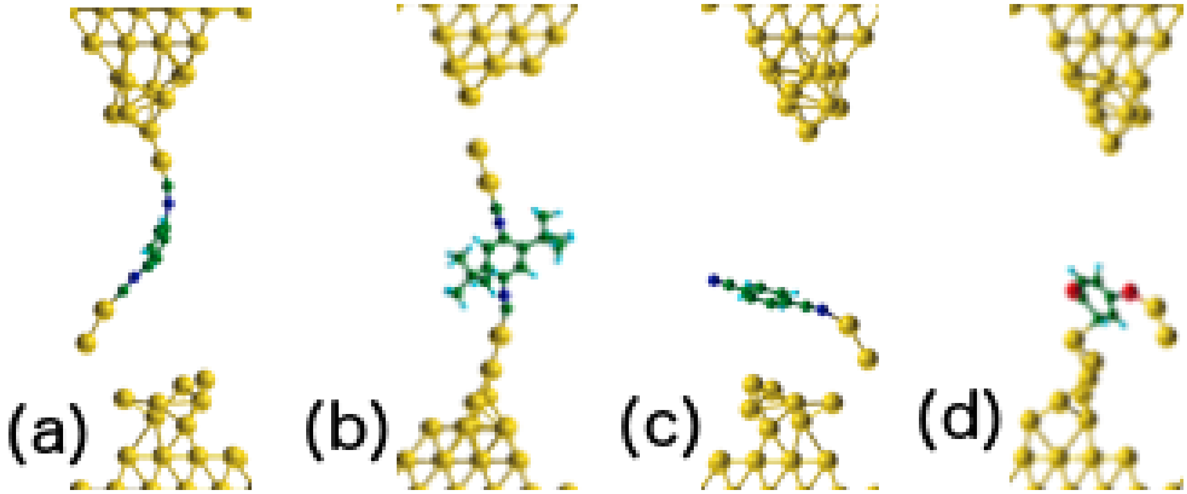

Supplementary Figure 9: Typical junction configurations after the breakage stage of an opening cycle for (a) BdNC; (b) tBuBdNC; (c) BdCN and (d) BdT.

We discuss now our ab-initio RTMD opening cycles, where we start from an arrangement where the molecule is attached to the pyramid tips at both electrode. We have found that BdNC junctions take always more length-steps to break than BDT- and especially than BdCN-junctions. Furthermore, after junction breaking, BdNC tends to point straight away from the pyramid and looks rather stiff. tBu-BdNC molecules behave similar to BdNC. BdCN junctions break very easily, while the stability of BDT junctions lies in between

the other two. After breakage, BDT is attached to one of the tips, but looks twisted. Supplementary Figure 9 shows typical configurations reached after junction breaking, and summarizes the above discussion. The figure also indicates that BdNC, tBuBdNC and BDT pull gold atoms before breaking, and that the junctions actually breaks after this second gold atom. We have seen this behaviour in all the BdNC and tBuBdNC opening cycles that we have simulated. In contrast, we have found that BDT junctions break half of the times at the molecule-tip bond. We also performed ab-initio RTMD cycles where we start closing a junction until a gold neck is formed, followed by an opening cycle whereby the gold neck breaks again leaving a single-molecule junction that eventually breaks upon further pulling. We always find that BdNC has a strong affinity to pull gold atoms.

Supplementary Note 10: Chaining effects

The above discussion hints that BdNC molecules may show a strong tendency to make Au-BdNC-Au-BdNC-Au supra-molecular complexes. We have therefore sought to test whether BdNC makes chains mediated by gold atoms. We have explored first quite extensively a simple gold-chain junction model, where we could easily and quickly make opening and closing cycles. We have first attached one BdNC/tBuBdNC/BdCN/BdT molecule to each of the two gold chains on a broken junction and have closed it. We have found that a junction never forms because the two molecules repel each other. In contrast, when we have attached gold atoms to the end of one of the molecules, tBuBdNC and BdNC molecules may form a molecule-gold-molecule dimer in many of the simulations. Furthermore, we have found that this bond is so strong that when we execute an opening cycle afterwards, the junction never breaks at the molecule-Au-molecule link, but at one of the electrode contacts instead. Interestingly, the molecule at the broken link pulls gold atoms also so that it is ready to make a trimer bdNC or tBuBdNC chain next.

To test this scenario more seriously we have next performed ab-initio RTMD simulations of closing/opening cycles using the more realistic (111) electrodes terminated by pyramids, where we start by a broken junction where a preformed dimer BdNC-Au-BdNC is attached to one of the two pyramids but not to the other. We have found that if the dimer is not terminated by gold, then it does not attach to the other electrode upon closing. Instead it bends and the free end drifts away from the gold neck that is eventually formed. We

have carried out 2 full closing/opening cycles where the dimer is terminated by gold. We have found in both cases that the dimer attaches to the other electrode and bends as the junction is closed until it becomes destroyed when the gold neck is formed, as we show in Supplementary Figure and Supplementary Figure . We then start pulling and break the gold neck. In one of the two simulations, the dimer reforms, while it does not reform in the other simulation. We have also performed 28 simulations of opening cycles, where 2 or 3 BdNC gold-capped molecules where attached to a gold neck. We found that a junction was formed in 14 of them. Five of these junctions contained a BdNC-gold-BdNC dimer, and one more contained a monomer in parallel with a dimer.

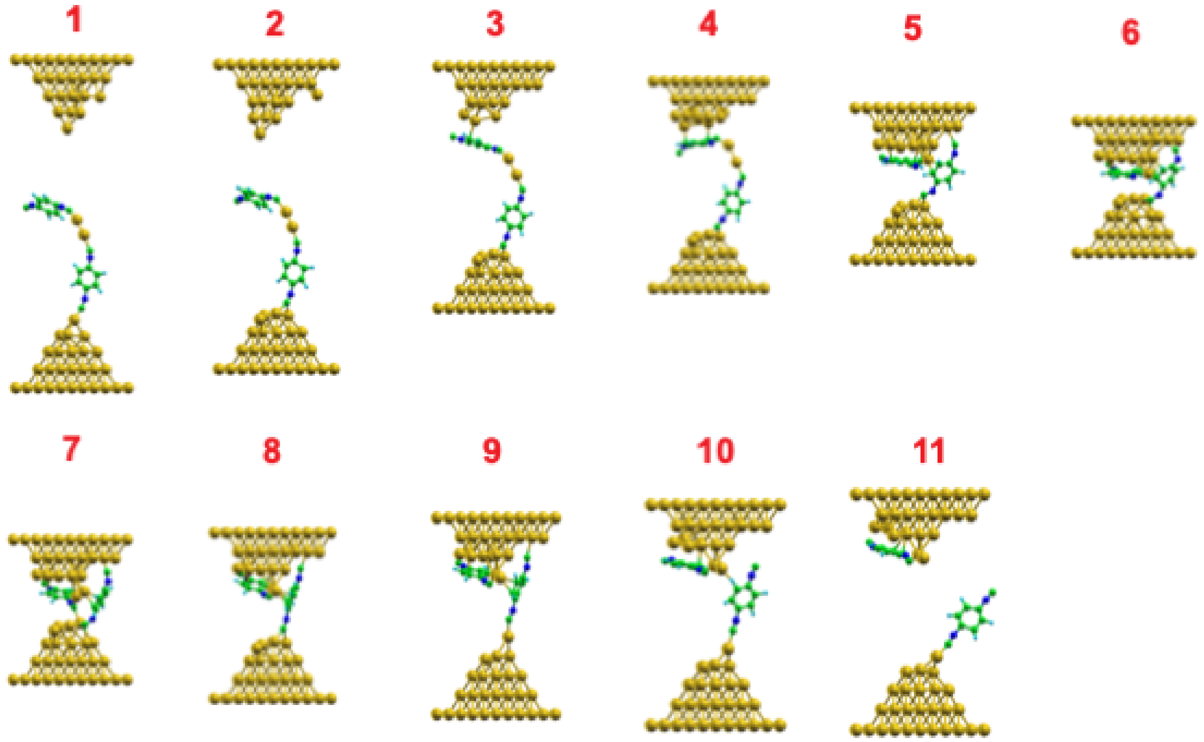

Supplementary Figure 10: Snapshots of a full closing/opening MD cycles, where a BdNC dimer is attached to the bottom electrode. The dimer is destroyed when the gold neck is formed and does not reform during the opening.

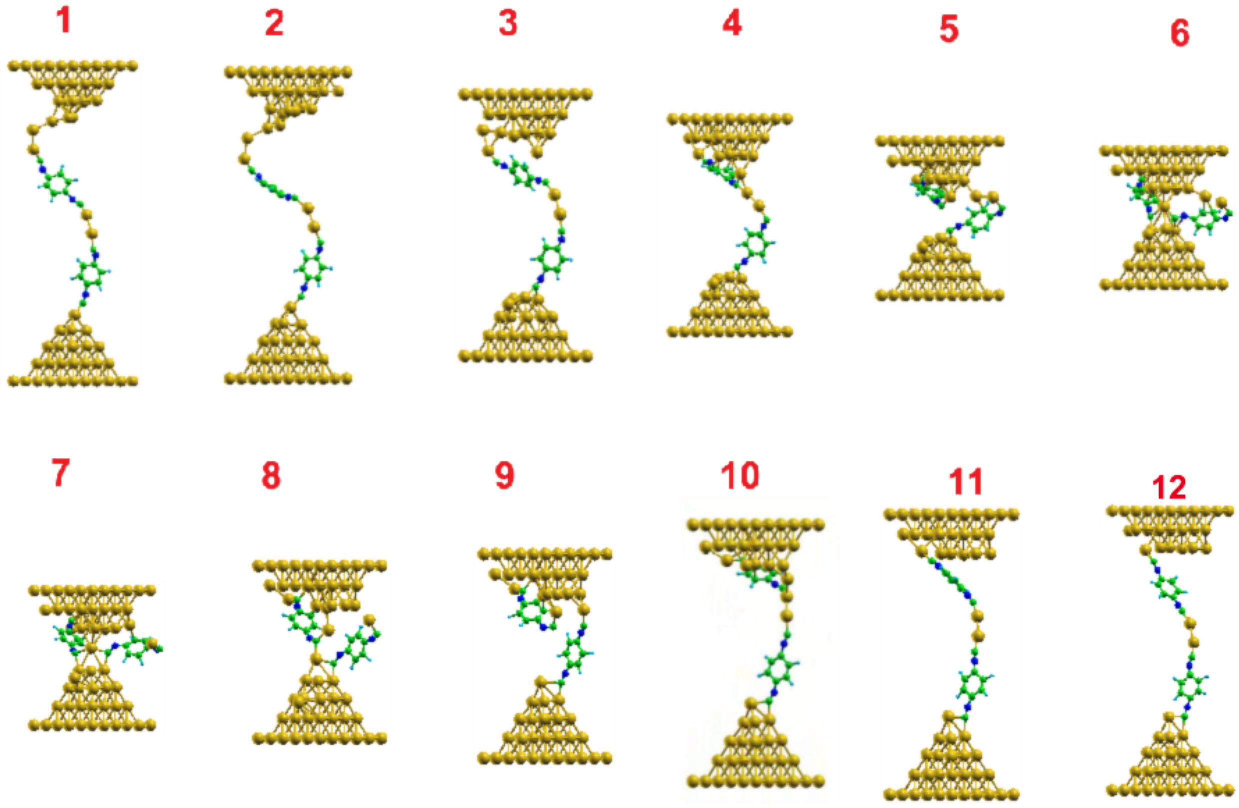

Supplementary Figure 11: Snapshots of a full closing/opening MD cycles, where a BdNC dimer is attached to the bottom electrode. The dimer is destroyed when the gold neck is formed and does reform during the opening.

We have also carried out a few MD simulations of closing traces for tBuBDT molecules to check whether they could bind to each other. Supplementary Figure shows snapshots of those traces. We have found that these molecules also bind to each other if they are gold-capped, but repel each other in they are uncapped. However, steric hindrance by the side groups restrict the probability that the tips of two gold-capped tBuBdNC molecules face each other at appropriate orientations and distances, so that the probability of tBuBdNC chain formation is very low.

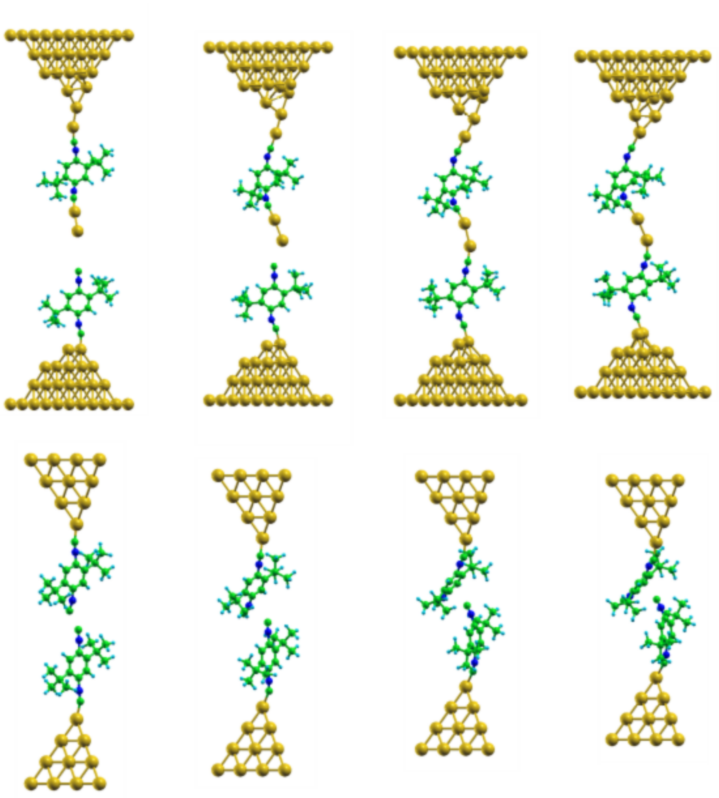

Supplementary Figure 12: Snapshots of two closing MD cycles, where two tBuBdNC molecules are attached to gold electrodes. The top panel shows that this molecules can bind to each other if they are gold-capped. The bottom panel shows that they do not bind if uncapped, rather, they repel each other.

This whole set of MD simulations sheds compelling evidence that BdNC molecule have a strong tendency to pull atoms from gold electrodes when stretched, and that they form molecular chains ..gold-BdNC-gold-BdNC-gold-.... We have also found that BdNC molecules bind to each other only by mediation of gold atoms. We have found that tBu-BdNC could also form gold-mediated chains but that the formation probability is rather low due to steric hindrance effects.

Supplementary Note 11: Scissor corrections scheme

DFT calculations deliver a wrong description of the Molecular Orbitals (MO) in a junction. Specifically, the HOMO-LUMO gap is under-estimated and the Fermi energy is not

well-placed. An opposite effect that also takes place in a junction is originated by screening of the electronic structure of the molecule by the conduction electrons in the electrodes. This effect shields the electron-electron interaction, effectively moving the MO closer to the Fermi energy, and depends inversely on the distance between the electrodes and the MO. A well-established way to remedy this failure is by performing a GW calculation. However, this sort of calculations are too computationally demanding, so it is really impossible to simulate a junction with a realistic number of atoms, specially if ab-initio MD simulations are required. The so-called Scissor Correction Scheme (SCS) is a phenomenological procedure that may remedy the above-described DFT failure. It consists of computing the Electron Affinity ($EA = E_0(N+1) - E_0(N)$) and Ionization Potential ($IP = E_0(N) - E_0(N-1)$) of the molecule in vacuum in terms of the corresponding ground-state energies E_0, that should be estimated correctly via DFT methods. Then the DFT HOMO and LUMO are repositioned accordingly.

$$\epsilon_{\mathrm{HOMO}} = IP = \epsilon_{\mathrm{HOMO,DFT}} - \Delta\epsilon_{\mathrm{HOMO}} \quad (2)$$

$$\epsilon_{\mathrm{LUMO}} = EA = \epsilon_{\mathrm{LUMO,DFT}} + \Delta\epsilon_{\mathrm{LUMO}} \quad (3)$$

SCS also takes care of the electrodes screening by further shifting ϵ_{HOMO} upwards and ϵ_{LUMO} downwards by an energy amount $\epsilon_{IC} = \frac{e^2}{8\,\pi\epsilon_0}\frac{\ln 2}{d} = \frac{13.6\,\ln 2}{d}$ eV. This estimate is taken from an image charge model where the electrodes are replaced by flat planes and the molecule by a point charge separated a distance d Å. Another even more phenomenological scheme is to shift ϵ_{HOMO} and ϵ_{LUMO} by whatever quantity is needed to fit the experiments. We have computed conductance traces and histograms of BdNC junctions by either not performing any shift at all (bare DFT), or by following the SCS scheme, and benchmarked both sets of results against experimental data. We have found that the SCS scheme provides rather good agreement with the experiments discussed in this article, while the bare DFT approach does not. The computed SCS shifts can be found in Supplementary TableIV.

Molecule	$\Delta\epsilon_{\mathrm{HOMO}}$ (eV)	$\Delta\epsilon_{\mathrm{LUMO}}$ (eV)
BdNC	2.6	2.5
BdNC dimer	2.3	1.25
tBu-BdNC	1.4	1.37

Supplementary Table IV: Shifts of the HOMO and LUMO levels according to the scissors corrections scheme

Supplementary Note 12: Statistics of the opening cycles

	Monomer	Dimer	Total
1 mol	2	0	2
2 mols	2	4	6
3 mols	7	1	8
Total	1	5	16

Supplementary Table V: Statistics of the opening cycles.

Supplementary Table V summarizes the different behaviours observed in the 30 opening cycles which have been performed, namely a single monomer, multiple monomers in parallel, and a dimer.

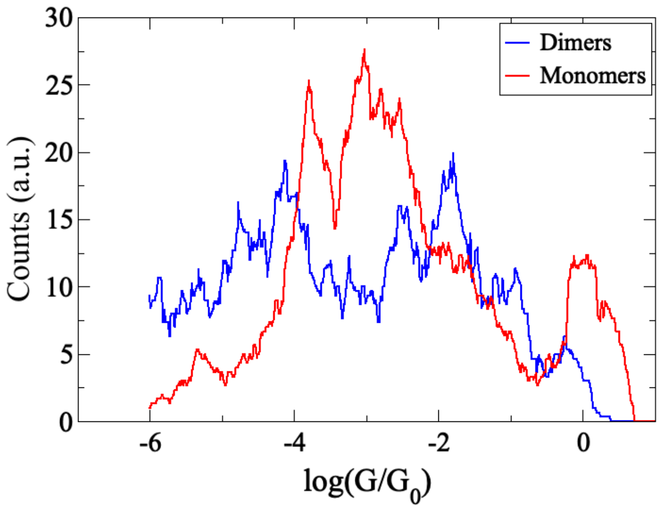

Supplementary Figure 13: Conductance histogram obtained for the (a) monomers, and (b) dimers.

Supplementary Figure 13 presents the conductance histograms constructed for the two different binding geometries. We find that the histogram of the dimers agrees well with the experimental values of P1 and P2 of BdNC at high concentration. Moreover, the conductance histogram for the simulated monomers resembles the experimental one for BdNC at low molecule concentration.

Supplementary Note 13: Decay constant for a chain with two interconnecting gold atoms

Supplementary Figure 14a presents three different junction geometries used to compute the decay constant in the case 2 gold are inserted in the chain, namely a monomer, a dimer and a trimer. Supplementary Figure 14b shows the computed transmission as a function of the electron energy, with respect to the Fermi energy of the gold electrodes.

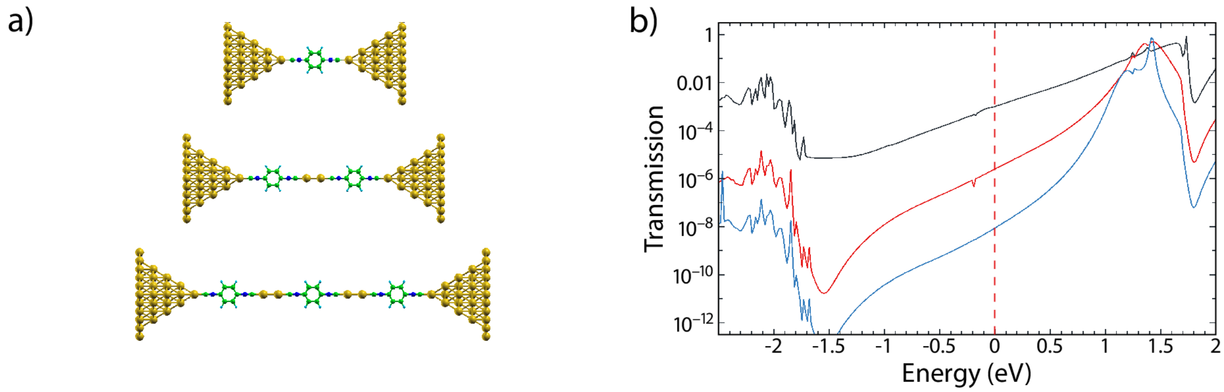

Supplementary Figure 14: (a) Junction geometries used to computed transmissions shown in b). (b) Computed transmission curves.

Supplementary References

[1] Soler, J. M. *et al.* The siesta method for ab initio order-n materials simulation. *J. Phys.: Condens. Matter* **14**, 2745 (2002).

[2] Buimaga-Iarinca, L. & Morari, C. Adsorption of small aromatic molecules on gold: a DFT localized basis set study including van der waals effects. *Theoretical Chemistry Accounts* **133**, 1502 (2014).

[3] Perdew, J. P. & Zunger, A. Self-interaction correction to density-functional approximations for many-electron systems. *Phys. Rev. B* **23**, 5048 (1981).

[4] Perdew, J. P., Burke, K. & Ernzerhof, M. Generalized gradient approximation made simple. *Phys. Rev. Lett.* **77**, 3865 (1996).

[5] Dion, M., Ridberg, H., Schroder, E., Langreth, D. C. & Lundqvist, B. I. Van der waals density functional for general geometries. *Physical Review Letters* **92**, 246401 (2004).

[6] Ferrer, J. *et al.* Gollum: a next-generation simulation tool for electron, thermal and spin transport. *New J. Phys.* **16**, 093029 (2014).

[7] Paulsson, M., Krag, C., Frederiksen, T. & Brandbyge, M. Conductance of Alkanedithiol Single-Molecule Junctions: A Molecular Dynamics Study. *Nano Letters* **9**, 117–121 (2009).
